# Most Italians attending a congress on health of elderly people do not know and do not recognize respiratory diseases

**DOI:** 10.1186/s40248-016-0062-3

**Published:** 2016-07-05

**Authors:** Nicola Ciancio, Claudio M. Sanguinetti, Franco Falcone, Claudio Taranto, Roberto Fasani, Fernando De Benedetto, Onofrio Resta, Fausto De Michele, Roberto Messina, Andrea Rossi, Stefano Nardini, Giuseppe Di Maria

**Affiliations:** Respiratory Physiopathology Study Group, Italian Society of Respiratory Medicine (S.I.Me.R.), Milan, Italy; Multidisciplinary Respiratory Medicine Official Journal of AIMAR, Borgomanero(NO), Italy; Dipartimento per i Rapporti Istituzionali e le Politiche Sanitarie in Pneumologia, Italian Association of Hospital Pneumologists (A.I.P.O.), Milan, Italy; Centro Studi S.I.C. Sanità In Cifre, FederAnziani Senior Italia Association, Rome, Italy; Clinical Research Organisation (CRO) Medi Service, Agrate Brianza, MB Italy; AIMAR (Interdisciplinary Association for the study of Respiratory Diseases), Arona (NO), Italy; Università degli Studi “Aldo Moro”, Bari, Italy; Italian Association of Hospital Pneumologists (A.I.P.O.), Milan, Italy; FederAnziani Senior Italia Association, Rome, Italy; Italian Society of Respiratory Medicine (S.I.Me.R.), Milan, Italy; Pneumology Unit, Policlinico Hospital, Via Santa Sofia, 78 95123 Catania, Italy

**Keywords:** COPD, Elderly people, Predictive symptoms, Respiratory health, Smoking, Spirometry

## Abstract

**Background:**

The present study reports the results of a survey jointly carried out by three Italian respiratory scientific associations (AIMAR, AIPO, SIMeR) together with an important Federation of elderly patients (FederAnziani) during the National Conference of Italian Court for Health Right held in Rimini from November 29^th^ to December 1^st^, 2013. The survey, based on a spirometric examination preceded by a questionnaire on respiratory health, was conducted on elderly people coming from all Italian regions to attend the Conference.

**Methods:**

Nine hundred forty-nine subjects (574 females and 375 males), mean age 66.2 ± 10.1 years, were interviewed and performed spirometric examination. There were 137 smokers (14.4 %). Mean value of *Body Mass Index* (BMI) was significantly higher in males (27.6 ± 6.6) than in females (26.3 ± 4.3).

**Results:**

17.1 % (*N* = 143) of the studied subjects reported to be suffering from respiratory disease and the prevalent illnesses were asthma (31.5 %) and COPD/emphysema (24.5 %), but only 3.3 % of the whole surveyed group was able to identify COPD as a pulmonary disease, however without knowing its characteristics, while these were known by 0.5 % of the interviewed subjects only.

A high number of subjects, 22 % of whom were smokers, declared chronic sputum production. 10.2 % of the study group showed an obstructive defect at spirometry when the criterium of lower limit of the normal (LLN) was considered, whereas it was 12.4 % if the fixed limit of 0.70 was chosen. 64 % of the obstructed people thought they did not have any respiratory disease.

**Conclusions:**

The results of this survey, able to spread the knowledge of respiratory diseases and spirometry in a wide sample of subjects for the most part scarcely aware of them, emphasize the need for a greater divulgation of respiratory issues among the general population.

## Background

Respiratory diseases represent the third cause of death in 28 states members of the European Community and among them chronic obstructive pulmonary disease (COPD) is the commonest one [1]. COPD is a heterogeneous condition, including chronic bronchitis, pulmonary emphysema, and small airways disease, very often associated with extra-pulmonary complications and comorbidities which can increase the disease severity. It is also characterized by a poorly reversible and progressive airflow limitation associated with an airway inflammation in response to noxious agents, mainly the cigarette smoke [2, 3]. COPD is one of the main causes of disability-adjusted life years (DALY) and its responsibility will increase worldwide by 2030 according to World Health Organization (WHO) [4, 5]. COPD patients refer a lower quality of life compared to the normal and to healthy subjects of the same age [6]. Moreover, COPD is the only chronic disease where a decreased life expectation has been recorded (mortality increased 163 %, whereas mortality for cardiovascular diseases varies from 35 % to 64 %). As a consequence, COPD still imposes a huge sanitary, social and economic burden and in Europe the indirect and direct costs of this disease are estimated as high as 38.7 billions euro [7]. In Italy the annual cost of COPD, including 2 or 3 medical consultations/year [8], is 2,110 euros per person and it almost doubles in case of hospital admission due to exacerbation [9].

In spite of these facts, COPD is still rather unknown and neglected not only by patients, but also by public health institutions. Many studies also demonstrated that patients can complain of symptoms but they frequently do not refer to their general practitioner [10, 11], especially in the initial phase of the disease, so that COPD is recognized when it has already reached a moderate/high degree of severity [12–14]. Thus, it seems really important to reach an early diagnosis in order to avoid the disease progression towards more severe and disabling stages, by means of a correct assessment of symptoms and a spirometric examination. However, spirometry is still underused not only in Italy [15], but also in other countries, like Sweden where only 30 % of patients diagnosed suffering from COPD presented the results of a spirometric examination in their health records [16], or in U.S.A. where only 31 % of COPD patients admitted to hospitals of care had performed a spirometric examination, while 78 % of those suffering from heart failure had performed an echocardiography [17]. Spirometry is poorly utilized to diagnose COPD also at general practitioners (GPs) level [18, 19] likely due to a difficulty in performing and interpreting the examination, to costs, availability of services, long wait times for the examination to be performed, and so on [20, 21].

In Italy there are not certain and updated estimates of COPD prevalence, that is evaluated around 4.5 % [22] but this is certainly an underestimation, as it is in other countries [23], thus it seems compelling to acquire more reliable data. With this aim the European Lung Foundation (ELF) and the European Respiratory Society (ERS) during their annual congresses from 2004 to 2009 organized the execution of spirometric examinations to reveal early obstructive defects in general population and recognize individuals at risk of developing COPD.

On a similar aim the survey devised and carried out by the three Italian scientific respiratory societies (AIMAR, Interdisciplinary Association for the study of Respiratory Disease; AIPO, Italian Association of Hospital Pneumologists; SIMeR, Italian Society of Respiratory Medicine) together with the Federation of elderly people (Federanziani) was based, whose results are reported hereinafter.

## Methods

Health Forum is an initiative promoted by FederAnziani and carried out during the National Congress of Court of Popular Justice for Health Right held in Rimini from November 29^th^ to December 1^st^, 2013. Among various health projects, the above respiratory scientific societies developed a project aimed at knowing respiratory diseases, including a questionnaire and a spirometric examination, addressed to elderly people coming from different Italian regions to attend the Congress. The questionnaire included personal data, smoking habit, clinical history with particular interest to respiratory diseases, presence of predictive symptoms, and level of knowledge of respiratory issues, mainly of asthma and COPD. Spirometries were performed with Spirolab III (Medical International Research srl, Rome, Italy), whose characteristics are in accordance with the ERS/ATS standards [24]. The spirometric examination was performed with the subject sitting and wearing a nose-clip. An obstructive defect was defined when FEV_1_/FVC (forced expiratory volume in one second/forced vital capacity) value was below the lower limit of normal (LLN) [25]; however also the fixed limit FEV_1_/FVC < 0.70 was used to define the presence of obstruction due to the worldwide use of this index especially in epidemiological studies. Based on FEV_1_ value as percent of the predicted (% pred) the obstructive disease was classified in different stages: stage I (FEV_1_ ≥ 80 % pred); stage II (FEV_1_ < 80 % pred ≥ 50 %) ; stage III (FEV_1_ < 50 % ≥ 30 %); and stage IV (FEV_1_ < 30 % pred) [26].

Statistical analysis was performed with MedCalc Software version 11.3.0.0 (Acacialaan 22, B-8400 Ostend, Belgium). Chi-square test was utilized to compare probability distribution of variables in the analyzed categories, and the test ANOVA to compare the variability into the different groups with that among the groups. Data are expressed as mean values. To evaluate the influence of risk factors a multinomial regression analysis has been conducted utilizing age decades, smoking habit, (current smokers, ex-smokers, never smokers), gender, and incidence of respiratory disease as independent variables, and LLN with the different severity stages (I to IV) as dependent variable.

## Results

### Characteristics of the study group

The study group includes 949 subjects (574 females (60.5 %) and 375 males (39.5 %), with a mean age of 66.2 ± 10.1 years (*range* 22–89) for the whole group ; mean age of females (65.3 ± 10.5 years, *range* 22–88) was significantly (*p* < 0.001) lower than that of males (67.7 ± 9.2, *range* 26–89). There were 339 (35.7 %) subjects less than 65 years old, 242 (71.4 %) females and 97 (28.6 %) males.

Mean value of *Body Mass Index* (BMI) was significantly (*p* < 0.001) higher in males (27.6 ± 6.6; *range* 18.6–43.3) than in females (26.3 ± 4.27 ; *range* 16.8–43.6) (Table 1).

**Table 1:** Demographic characteristics of the studied sample

	Females *N* = 574	Males *N* = 375	Total *N* = 949	*p*
Age	65.3	67.7	66.2	*<0.001*
Standard deviation	10.52	9.18	10.08	
Median	66.0	68.0	67.0	
Min - max	22–88	26–89	22–89	
Age < 65 years	242 (42.2 %)	97 (25.9 %)	339 (35.7 %)	*<0.001*
Age ≥ 65 years	332 (57.8 %)	278 (74.1 %)	610 (64.3 %)	
BMI	26.3	27.6	26.7	*<0.001*
Standard deviation	4.27	6.60	5.87	
Median	25.9	26.6	26.3	
Min - max	16.8–43.6	18.6–43.3	16.8–43.6	

### Results of the questionnaire

**Fig. 1:**
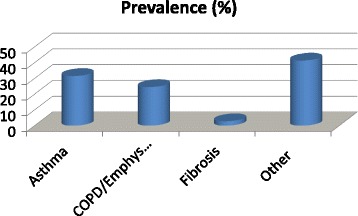
Prevalence of the reported respiratory diseases in the study group

837 subjects, 301 (36.0 %) aged < 65 years, 536 (64.0 %) > 65 years, answered the question whether they were suffering from respiratory disease and 143 (17.1 %) (*n* =143) gave a positive reply, while among subjects aged less than 65 years, 46 (15.3 %) answered to be suffering from respiratory diseases, of which 60 in total were taking drugs. The main reported respiratory diseases were asthma (*n* = 45; 31.5 %); COPD/emphysema (*n* = 35; 24.5 %) and pulmonary fibrosis (*n* = 4; 2.8 %) (Fig. 1).

There were 523 (55.1 %) non smokers, 289 (30.5 %) ex-smokers, and 137 (14.4 %) current smokers, mean of 14.4(±9.6) cigarettes/day, mean smoking duration 31.5 (±14.3) years. The majority of current smokers had tried to give up smoking at least once, 75 % of them by oneself, 10.3 % turning to a center for smoking cessation, 4.4 % assisted by their general practitioner, and 10.3 % in a different way.

632 subjects answered the question about COPD knowledge, and 459 (72.6 %) out of them affirmed not to know the disease, 149 (23.6) only knew the name of the disease, 21 (3.3 %) were aware that COPD is a respiratory disease but were not able to describe the characteristics of the disease, while only 3 subjects (0.5 %) revealed a detailed and comprehensive knowledge of the disease. The percentage of those who had a generic or detailed knowledge of COPD was higher in the group suffering from respiratory diseases (*p* = 0.044). The results of the questionnaire concerning the presence of predictive respiratory symptoms are shown in Table 2, where the number of subjects reporting the presence of chronic sputum production (22.4 % of whom were current smokers) was very high.

**Table 2:** Distribution of answers to questionnaire concerning predictive symptoms of respiratory disease

Symptoms of respiratory disease	YES	NO	% Positive answers
Do you cough frequently? (796 answers)	173	623	27.8
Do you frequently have sputum production with cough? (802 a.)	281	521	53.9
Do you complain of dyspnea under mild efforts? (808 a.)	278	530	52.4
During physical activity do you have more shortness of breath than your companions of the same age? *N* = 764	278	496	56
Do you perceive wheezing in your chest when you breathe under effort? (792a.)	113	679	16.6
Do you perceive wheezing in your chest when you breathe at night? (599 a.)	69	530	13
Do you frequently catch a cold lasting more than that of other people you know? (811 a.)	186	625	29.8

### Spirometry results

Data of 948 spirometries were examined : 85 subjects (10.2 %) presented a FEV_1_/FVC rate lower than LLN, and 58 out of them thought they were not suffering from any respiratory disease (here indicated as “false healthy” subjects). Among the subjects who knew to be suffering from broncho-obstructive disease (FEV_1_/FVC < LLN), 14 (51.9 %) had FEV_1_ > 80 % of the predicted value, 11 (40.7 %) FEV_1_ between 79 and 50 % pred, and 2 (7.4 %) FEV_1_ < 50 % pred. Among the “false healthy” subjects 45 (77.6 %) had FEV_1_ > 80 % pred, 13 (22.4 %) FEV_1_ between 79 and 50 %. The results relative to disease severity with both methods of measure are reported in Tables 3 and 4.

**Table 3:** Results of spirometric examination: number of subjects with airways obstruction using the fixed value of 0.70 or the LLN

	FEV_1_/FVC <0.70	FEV_1_/FVC < LLN
Tot.	104	85
“False Healthy”^a^	67	58
Patients^b^	37	27

^a^”False healthy” = Subjects with obstructive defect who thought to be healthy

^b^Patients = Subjects with obstructive defect aware to be suffering from respiratory disease

**Table 4:** Results of spirometric examination: subjects distribution in relation to severity of obstruction and method of obstruction assessment

	FEV_1_/FVC < LLN “False healthy”^a^	FEV_1_/FVC < LLN Patients^b^	FEV_1_/FVC < 0.70 “False healthy”^a^	FEV_1_/FVC < 0.70 Patients^b^
FEV_1_ ≥ 80 %	45	14	52	22
FEV_1_ < 80 ≥ 50 %	13	11	15	13
FEV_1_ < 50 %		2		2

^a^”False healthy” = Subjects with obstructive defect who thought to be healthy

^b^Patients = Subjects with obstructive defect aware to be suffering from respiratory disease

Forced vital capacity was not influenced by smoking habit in our study group (mean value 104.0 ± 25.5 % pred in non smokers + ex-smokers ; 101.4 ± 22.5 % pred in current smokers; *p* = not significant), and it was the same when the presence of respiratory disease was taken as discriminant. On the contrary, mean percent value of FEV_1_ was significantly reduced in current smokers (97.0 ± 20.9 % pred) compared to non smokers + ex-smokers (102.9 ± 22.1 % ; *p* = 0.004) and in relation to the presence of respiratory disease (*p* < 0.001). Similarly, the value of mean expiratory flow was negatively influenced by smoking habit and presence of respiratory disease. Comparing predictive symptoms results and spirometric data, the percentage of subjects complaining of frequent cough, chronic sputum production, and wheezing also at night was more elevated among those with FEV_1_/FVC < LLN (Table 5). Applying a logistic regression model to individuate predictive symptoms correlated with the obstructive defect in the group of “false healthy” subjects, the variables significantly correlated with the dichotomous index < or > LLN were: age (mean age lower in subjects with FEV_1_/FVC < LLN, productive cough (46.8 % in those with the rate lower than LLN and 26.8 % in those with the rate greater than LLN); wheezing under effort (6.6 % in < LLN and 9.0 % in the others), and wheezing at night (11.5 % in < LLN and 5.8 % in the other group). The percentage of subjects complaining of productive cough was significantly higher (*p* = 0.002) in obstructed subjects compared to the non-obstructed ones.

**Table 5:** Predictive symptoms of respiratory disease in relation to the presence of obstructive defect at spirometry

Symptoms of respiratory disease	Percentage of subjects answering yes to the question		*p*
	FEV_1_/FVC < 70 or < LLN	Normal spirometry results	
Do you cough frequently?	*N* = 108	*N* = 687	*0.008*
	34 (31.5 %)	139 (20.2 %)	
Do you frequently have sputum production with cough?	*N* = 113	*N* = 688	*0.002*
	54 (47.8 %)	226 (32.8 %)	
Do you complain of dyspnea under mild efforts?	*N* = 113	*N* = 694	*0.368*
	43 (38.1 %)	234 (33.7 %)	
During physical activity do you have more shortness of breath than your companions of the same age?	*N* = 109	*N* = 654	*0.177*
	46 (42.2 %)	232 (35.5 %)	
Do you perceive noises in your chest when you breathe under effort?	*N* = 115	*N* = 676	*0.029*
	28 (20.9 %)	89 (13.2 %)	
Do you perceive noises in your chest when you breathe at night?	*N* = 92	*N* = 506	*<0.001*
	21 (22.8 %)	48 (9.5 %)	
Do you frequently catch a cold lasting more than that of other people you know?	*N* = 114	*N* = 696	*0.065*
	34 (29.8 %)	153 (22.0 %)	

## Discussion

The chronic and disabling diseases, among which COPD holds a prominent position, are a serious social and economic problem; in fact their incidence is increasing and they are responsible for more than 35 millions of deaths all around the world, also causing a relevant amount of life years with disability conditions [27]. In Italy, respiratory diseases represent the third cause of death and their prevalence is estimated to increase also due to the population’s aging. Asthma prevalence is higher in females at all ages except for the interval 15–34 years, while BPCO is prevalent in males at all age ranges and this is more evident in patients over 64 years old [28]. In our study group, mainly composed of elderly people, the prevalence of respiratory diseases is more than 17 % and the most frequent diseases are asthma and COPD in accordance with the above mentioned national data. The increase in chronic respiratory diseases is also influenced, besides age, by the risk factors that sustain these diseases. Direct and indirect costs of chronic diseases and their consequences exert a significant weight on budget of industrialized countries. Likely, a widespread knowledge of the basic issues of a certain disease would contribute to reduce its incidence, to reach the diagnosis earlier, and to increase patients’ adherence to treatment.

The aim of the present survey was to spread the knowledge of respiratory diseases, COPD in particular, in a wide random sample of elderly people coming from different Italian regions to attend the Health Forum annually promoted by FederAnziani in Rimini.

Questionnaires and spirometric examinations yielded interesting results and, if some well established concepts are confirmed, notably important elements are suggested for further intervention aimed at diffusing the knowledge of chronic respiratory diseases and improve their diagnosis.

In this respect, the answers to the question concerning the meaning of the COPD acronym and the knowledge of the characteristics of this disease are very significant. In fact, only 3 out of 632 interviewed subjects were aware of COPD characteristics and other 15 subjects were able to clearly define COPD as an obstructive airways disease. This, likely, indicates a lack or insufficiency of communication concerning the characteristics of COPD, a potentially disabling and highly resource-consuming disease, among the general population. A campaign aimed at sensitizing population through audiovisual means of communication is at least advisable to make people understand the importance of abolishing risk factors and reaching an early diagnosis in order to reduce COPD incidence and its progression.

The main predictive symptoms reported in this study are chronic sputum production and dyspnea also under minimal efforts. COPD generally presents with persistent cough, continuous sputum production and dyspnea but these symptoms are often overlooked especially by smokers at initial stages of the disease, because they do not consider these symptoms as a marker of illness but only an unavoidable consequence of smoking. Thus, the diagnosis is delayed and obtained when the disease already  has reached greater severity with important and irreversible pathologic and functional consequences and increased social and economic burden.

It is well established that cigarette smoking is a main cause of COPD origin, and the etiologic COPD fraction attributable to smoking varies from 70 % to 80 % in several studies [29]. In the present survey only 137 subjects (14.4 % of the 949 interviewed subjects) declared to be current smokers. This prevalence of smokers is lightly higher than that recorded on the average in Italian population of the same age. In fact, data published this year by the Italian Observatory for Smoking, Alchol, and Drug (OSSFAD) report in general 20.8 % of smokers (25.1 % in men and 16.9 % in women), but a mean of 9.9 % (14.2 % in men and 6.7 % in women) in subjects 64 years old or more and 18.3 % in the age range 45–64 years [30]. Likely, the mean percentage of smokers is higher in our study group because more than one third of subjects were less than 65 years old.

The first reference for an effective action against smoking are GPs who should always record the smoking condition in every assisted patient, besides the amount of smoked cigarettes, and the duration of this practice. Indeed, this does not always happen because a wide inquiry at GPs’ level revealed that only 53 % of them know how many smokers or ex-smokers are in the whole group of patients they assist, and only about 30 % applied an even minimal counselling protocol to favor smoking cessation [21]. These data are in accordance with the fact that in the present study the majority of smokers who affirmed having tried to give up smoking did it by themselves, less than 5 % with the GP assistance, and about 10 % turning to a smoking cessation center. Furthermore, an intervention with greater effectiveness for adolescents to not start smoking and adult smokers to give up smoking is really essential to prevent not only respiratory diseases, but also other chronic diseases that can cause death and disability [31].

About 10 % of subjects who underwent spirometry in this study presented an obstructive defect of various entity and this can be related to the fact that asthma and COPD together are present in 10 % of the whole group. The incidence of airways obstruction in our patients is lower than that observed in the spirometry project on general population carried out by ERS where a 12.4 % of obstructed subjects using the LLN index and 20.3 % using the fixed ratio 0.70 were recorded. In several studies carried out by GPs especially in smokers, the incidence of obstructive defect at spirometry varies from 22.2 % in a Belgian study to 29.9 % in another investigation performed in Holland [32, 33]. However, what seems rather alarming is that 70 % of subject with airways obstruction in our survey thought they did not have any respiratory disease. This can be explained with the fact that the majority of them presented a mild or, in some cases, moderate obstructive defect, thus they gave no importance to the possible limiting effects of the obstruction or to the predictive symptoms, especially if smokers, and did not seek medical consultation neither had ever performed a spirometric examination.

Thus, our findings that confirm the negative influence of cigarette smoking on respiratory function make once more really urgent the need for organizing the fight against smoking at any level better than how already done.

In our study also the value of predictive symptoms is clearly confirmed by comparing the incidence of symptoms in subjects with airways obstruction and those without obstruction. Moreover, respiratory symptoms were present in 50.4 % of 125 smokers and in 34.7 % of 677 non smokers (*p* < 0.001). Presence of chronic cough and sputum production is significantly increased in the group of subjects with airways obstruction and the predictive value of disease and possible airways obstruction inherent to these symptoms, especially chronic cough, is confirmed also in the group of obstructed patients who thought to be healthy.

Presence of dyspnea for mild efforts in our sample of subjects does not seem to correlate with the respiratory function defect, at least with the simple measurement of FEV_1_/FVC; however it cannot be excluded the responsibility for the determinism of this symptom of cardiovascular diseases alone or associated with the respiratory ones, possibly revealable with more sophisticated functional examinations.

## Conclusions

Clinical and instrumental respiratory assessment of a wide sample of elderly population coming from all Italian regions allowed to point out some interesting aspects, first to highlight that the incidence of chronic respiratory diseases is very high (more than 17 %) in general population at this age. The negative role of smoking in terms of respiratory function defect and the predictive value of some key symptoms like chronic cough and sputum production were confirmed.

This survey has represented a unique opportunity to spread the knowledge of respiratory diseases and spirometric examination in a wide amount of subjects for the most part unaware of them, and the results emphasize the need to increase the divulgation of respiratory issues in order to improve life style, mainly in relation to smoking habit, and possibly reach an earlier diagnosis of respiratory disease when it would be still possible to slow up the structural alterations and the functional decline.
